# MicroDAIMON study: Microcirculatory DAIly MONitoring in critically ill patients: a prospective observational study

**DOI:** 10.1186/s13613-018-0411-9

**Published:** 2018-05-15

**Authors:** Claudia Scorcella, Elisa Damiani, Roberta Domizi, Silvia Pierantozzi, Stefania Tondi, Andrea Carsetti, Silvia Ciucani, Valentina Monaldi, Mara Rogani, Benedetto Marini, Erica Adrario, Rocco Romano, Can Ince, E. Christiaan Boerma, Abele Donati

**Affiliations:** 1 Anaesthesia and Intensive Care, Department of Biomedical Sciences and Public Health, Università Politecnica delle Marche, via Tronto 10/a, 60126 Ancona, Italy; 2 Department of Translational Physiology, Academic Medical Centre, University of Amsterdam, Meibergdreef 9, 1105 AZ Amsterdam, The Netherlands; 3 Department of Intensive Care, Medical Centre Leeuwarden, Henri Dunantweg 2, 8934 AD Leeuwarden, The Netherlands

**Keywords:** Microcirculation, Physiologic monitoring, Critical illness, Tachycardia, Video microscopy, Capillaries

## Abstract

**Background:**

Until now, the prognostic value of microcirculatory alterations in critically ill patients has been mainly evaluated in highly selected subgroups. Aim of this study is to monitor the microcirculation daily in mixed group of Intensive Care Unit (ICU)-patients and to establish the association between (the evolution of) microcirculatory alterations and outcome.

**Methods:**

This is a prospective longitudinal observational single-centre study in adult patients admitted to a 12-bed ICU in an Italian teaching hospital. Sublingual microcirculation was evaluated daily, from admission to discharge/death, using Sidestream Dark Field imaging. Videos were analysed offline to assess flow and density variables. Laboratory and clinical data were recorded simultaneously. A priori, a Microvascular Flow Index (MFI) < 2.6 was defined as abnormal. A binary logistic regression analysis was performed to evaluate the association between microcirculatory variables and outcomes; a Kaplan–Meier survival curve was built. Outcomes were ICU and 90-day mortality.

**Results:**

A total of 97 patients were included. An abnormal MFI was present on day 1 in 20.6%, and in 55.7% of cases during ICU admission. Patients with a baseline MFI < 2.6 had higher ICU, in-hospital and 90-day mortality (45 vs. 15.6%, *p* = 0.012; 55 vs. 28.6%, *p* = 0.035; 55 vs. 26%, *p* = 0.017, respectively). An independent association between baseline MFI < 2.6 and outcome was confirmed in a binary logistic analysis (odds ratio 4.594 [1.340–15.754], *p* = 0.015). A heart rate (HR) ≥ 90 bpm was an adjunctive predictor of mortality. However, a model with stepwise inclusion of mean arterial pressure < 65 mmHg, HR ≥ 90 bpm, lactate > 2 mmol/L and MFI < 2.6 did not detect significant differences in ICU mortality. In case an abnormal MFI was present on day 1, ICU mortality was significantly higher in comparison with patients with an abnormal MFI after day 1 (38 vs. 6%, *p* = 0.001), indicating a time-dependent significant difference in prognostic value.

**Conclusions:**

In a general ICU population, an abnormal microcirculation at baseline is an independent predictor for mortality. In this setting, additional routine daily microcirculatory monitoring did not reveal extra prognostic information. Further research is needed to integrate microcirculatory monitoring in a set of commonly available hemodynamic variables.

*Trial registration* NCT 02649088, www.clinicaltrials.gov. Date of registration: 23 December 2015, retrospectively registered

**Electronic supplementary material:**

The online version of this article (10.1186/s13613-018-0411-9) contains supplementary material, which is available to authorized users.

## Background

The microcirculation is a vast network of small vessels (terminal arterioles, capillaries and venules < 100 µm diameter) in which the exchange of oxygen and nutrients with tissues takes place [1]. Its derangement, defined as “microcirculatory shock” [2], is recognised as an important cause of organ dysfunction in critically ill patients, affected by various disease states, such as sepsis, severe trauma, haemorrhagic shock and post-cardiac arrest [2–5]. Furthermore, microcirculatory abnormalities and its persistence despite adequate macro-hemodynamic resuscitation were independently associated with morbidity and mortality in many critical conditions [6–12].

Today, the development of new technologies of in vivo video microscopy and its integration in easy-to-handle microscopes as in Sidestream Dark Field (SDF) imaging allow us to assess the (sublingual) microcirculation at the bedside, in a non-invasive way [13]. However, until 2015, data on microcirculatory alterations in the Intensive Care Unit (ICU) were restricted to small sample-sized studies in high-risk patients [7, 8, 14].

The MicroSOAP study by Vellinga and colleagues [15] gave a first insight in the *prevalence* of microcirculatory alterations in a large number of ICU patients. However, due to its design with a single time-point observation, the *incidence* in a time-dependent manner remains to be elucidated. Primary aim of the study was to detect a difference in the incidence of microvascular flow abnormalities between ICU survivors and non-survivors. Secondary outcomes were long-term mortality (in-hospital mortality and 90-day mortality) and development of organ dysfunction (described by sequential organ failure assessment, SOFA).

## Methods

### Patients enrolment and data collection

The MicroDAIMON (Microcirculation DAIly MONitoring in critically ill patients) is a single-centre prospective observational study (clinicaltrials.gov, NCT 02649088 registered on 23 December; retrospectively registered). The recruiting phase was performed in a 9-month period in 2013 (from 1 April to 31 December) in a 12-bed mixed ICU of an Italian teaching hospital with a mean number of yearly-admitted patients of 400. The ICU was structured in three subunits of four beds each, caring for respiratory, traumatology and medical critically ill patients, respectively. For the study purpose, each subunit was subsequently included and monitored during a 3-month period for patients’ screening and the recruitment: from 1 April to 30 June 2013, the medical subunit, from 1 July to 30 September 2013, the traumatology subunit and from 1st October to 31st December 2013, the respiratory subunit.

Patients were screened and included in the study within the first 12 h from ICU admission. Exclusion criteria were age < 18 years, lack of informed consent and pathophysiological conditions that may interfere with the sublingual microcirculation videos acquisition (maxillofacial traumas/surgery, oral bleeding, mucositis, etc.). In context to the microcirculatory assessments, demographic, laboratory, microbiologic, hemodynamic and other clinical data were recorded. All patients were followed up for 90 days after the ICU admission.

The study protocol was approved by the Local Ethics Committee and conducted in respect of the principles of Helsinki declaration (last revision, Edinburgh 2000). A written informed consent was obtained from all the included subjects or their next of kin in compliance with national applicable laws.

### Microcirculation assessment

The sublingual microcirculation was evaluated at the moment of the inclusion and every 24 h until discharge/death with SDF imaging (Microscan^®^, Microvision Medical, Amsterdam, The Netherlands) [13].

The video acquisition technique is extensively described in previous papers [16]. For every session, videos from at least five different sites were registered trying to obtain a good video quality and to avoid artefacts that may affect flow or vessels density variables [16].

The three best videos were chosen from each session, in compliance with recommendations from Massey et al. [17] and blindly analysed offline with a dedicated software (Automated Vascular Analysis, AVA Software 3.0, MicroVision Medical, Amsterdam, The Netherlands) by a restricted group of four experienced investigators. Inter-observer variability was calculated, based on the simultaneous analysis of ten randomly selected SDF videos by all the investigators. Variables of flow (Microvascular Flow Index, MFI and proportion of perfused vessels, PPV), as well as capillary density (total vessel density, TVD, perfused vessel density, PVD) and flow distribution (Heterogeneity Index, HI) were calculated according to international criteria [18, 19]. Flow was scored per quadrant as 0 (no flow), 1 (intermittent flow), 2 (sluggish flow) and 3 (continuous flow). The MFI is the average over 4 quadrants × 3 areas of interest. Total vessel density (TVD, mm/mm^2^) was calculated as the total length of vessels divided by the total area of the image. The percentage of perfused vessels (PPV) was estimated as follows: 100 ×  [(total number of grid crossings − [no flow + intermittent flow])/total number of grid crossings] and expressed as percentage. The perfused vessel density (PVD, mm/mm^2^) was estimated by multiplying TVD by PPV as estimated with the De Backer method. The Flow Heterogeneity Index (FHI, arbitrary units) was calculated as the highest MFI minus the lowest MFI, divided by the mean MFI of all sublingual sites [18].

Analogous to previous data, a threshold for the MFI < 2.6 was a priori established to define an abnormal microcirculation [3, 8, 15, 20].

### Statistical analysis

Data analysis was conducted with SPSS Software 17.0 (IBM, New York, NY) and GraphPad Prism 6 (GraphPad Software, La Jolla, CA). All data are presented as mean ± standard deviation (SD) or median [interquartile range, IQR].

Descriptive statistics were performed to obtain patients’ baseline characteristics. Quantitative variables distribution was tested with Kolmogorov–Smirnov normality test. Parametric (Student’s *t* test with Welch’s correction) and nonparametric tests (Mann–Whitney *U* test) were applied to describe the differences between groups for the variables of interest as appropriate. Fisher’s exact test was performed for comparisons between categorical variables, and the results are presented as percentage, odds ratio (OR) and 95% confidence interval (CI). Kaplan–Meier 90-day survival curves with Tarone–Ware test for the comparison of the hazard ratio between groups were built for the survival analysis.

Binary logistic regression analysis was performed with a forward stepwise entry method. A *p* value of less than 0.05 was considered statistically significant.

## Results

### Population characteristics

During the study period, 40, 37 and 38 patients were admitted, respectively, in the medical, traumatology and respiratory ICU subunits, for a total amount of 115 patients. Hundred patients met the inclusion criteria. All the patients were included in the study within 12 h from ICU admission, with no exceptions due to timing or organizational issues. Three patients were a posteriori excluded because no SDF videos were available for the baseline assessment. Therefore, 97 patients were included in the final analysis. The flow chart for the patients’ inclusion process is illustrated in Additional file 1.

Baseline characteristics of the patients are illustrated in Table 1. Patients were predominantly male (66%) with a median age of 67 years [46–75], a mean acute physiology and chronic health evaluation (APACHE) II score of 16 ± 7 and a median SOFA score of 7 [4–10]; the most frequent cause of ICU admission was trauma (38.1%). Patients admitted for sepsis represented the 9.3% of the sample. During the ICU stay, ten more patients developed sepsis: two trauma patients (5.4%), one neurologic patient (4.8%), two respiratory patients (18.2%) and five other patients (26.3%).

**Table 1:** Baseline characteristics and comparison between ICU survivors versus non-survivors

Patients characteristics	*n*	All (97)	ICU survivors (76)	ICU non-survivors (21)	*p*
Male gender (*n*, %)	97	64 (66)	50 (65.8)	14 (66.7)	1
Age (years, *n*)	97	67 [46–75]	64 [44–73]	71 [56–81]	0.034
APACHE II (pts)	97	16 ± 7	14 ± 7	22 ± 6	< 0.001
SOFA (pts)	97	7 [4–10]	6 [4–9]	12 [8–15]	< 0.001
ICU admission diagnosis, *n* (%)	97				0.046
		Trauma 37 (38.1)	34	3(8.1)	
		Neurologic 21 (21.6)	17	4(19)	
		Respiratory 11 (11.3)	8	3(27.3)	
		Sepsis 9 (9.3)	6	3 (33.3)	
		Other 19 (19.7)	11	8(42.1)	
Heart rate (bpm)	97	79 [61–102]	77 [61–95]	96 [69–107]	0.045
Mean arterial pressure (mmHg)	97	84 ± 19	86 ± 17	79 ± 27	0.255
Vasoactive drugs (treated)	54		38(50)	16(76.2)	0.046
*Noradrenaline (mcg/kg/min)*	52	0.28 [0.14–0.61]			
*Dopamine (mcg/kg/min)*	5	6.1 [4.8–7.4]			
*Dobutamine (mcg/kg/min)*	8	2.54 [2–4.5]			
Cumulative Vasopressor Index	54	4 [4]	1 [0–4]	4 [2–4]	0.008
Glasgow Coma Scale (pts)	97	10 [3–15]	10 [4–15]	4 [3–14]	0.074
Mechanical ventilation (*n*, %)	97	91 (93.8)	71(93.4)	21(100)	0.581
Peep (cmH_2_O)	91	7 [6–9]	7 [6–9]	8 [7–10]	0.095
Haemoglobin (g/dL)	97	11 ± 1.78	11.1 ± 1.7	10.8 ± 2.2	0.58
White blood cells (*n* × 10^3^/mmc)	97	12.1 [8.83–14.81]	11.3 [8.8–15.7]	12.7 [9.5–14.6]	0.518
Platelets (*n* × 10^3^/mmc)	97	150 [102–199]	165 [110–201]	115 [56–173]	0.02
Creatinine (mg/dL)	97	1.0 [0.8–1.45]	1 [0.8–1.2]	1.4 [1.1–1.8]	< 0.001
Bilirubine (mg/dL)	97	0.8 [0.5–1.2]	0.75 [0.5–1.1]	0.9 [0.4–1.8]	0.264
PaO_2_ (mmHg)	97	146 [104–175]	147 [106–176]	140 [93–172]	0.63
Arterial lactates (mmol/L)	97	1.4 [1.0–2.15]	1.3 [0.9–1.67]	3.1 [1.4–5.6]	< 0.001
ScvO_2_ (%)	60	77.2 [71–82.3]	77.7 [72.3–82.5]	75.6 [62.7–80.8]	0.24
*Microcirculatory variables*					
TVD (small) (mm/mm^2^)	97	20.4 ± 3.7	20.5 [17.2–22.7]	20.9 [17.8–22.7]	0.817
PVD (small) (mm/mm^2^)	97	19.3 ± 4.4	19.3 ± 4	19.3 ± 6	0.951
De Backer score (small) (n/mm)	97	11.9 ± 2	11.8 ± 2	12.2 ± 2	0.489
PPV (small) (%)	97	98.3 [95.4–100]	98.2 [94.8–100]	98.3 [97–100]	0.688
MFI (small) (AU)	97	3 [2.7–3.0]	3 [2.75–3]	2.93 [2.3–3]	0.155
HI (small)	97	0 [0.0–0.2]	0 [0–0.2]	0 [0–0.3]	0.417
Abnormal MFI (*n*, %)	97	20 (20.6)	11 (14.5)	9 (42.9)	0.012

Data are presented as mean ± or as median [IQR] unless stated otherwise

*APACHE* acute physiologic and chronic health evaluation II, calculated over the first 24 h from ICU admission; *SOFA* sequential organ failure assessment, calculated over the first 24 h from ICU admission; *CVI* cumulative vasopressor index; *ICU* Intensive Care Unit; *COPD*, chronic obstructive pulmonary disease. *TVD* total vessel density; *PVD* perfused vessel density; *PPV* proportion of perfused vessel; *HI* Heterogeneity Index; *MFI* Microvascular Flow Index. Abnormal MFI is defined as MFI < 2.6. Cut-off value for small vessels diameter < 20 μm

Median ICU length of stay was 7 [4–15] days; ICU mortality was 21.6%, in-hospital mortality 34%, 90-day mortality 31.9% (two patients died in the hospital after 90 days from ICU admission).

### Microcirculatory abnormalities at baseline and outcome

2455 videos were collected and analysed offline to obtain microcirculatory variables. The coefficient of variation (inter-observer variability) for MFI was 1.4 ± 3% for small vessels. Baseline microcirculatory variables are described in Table 1. The incidence of MFI abnormality at the day of ICU admission was 20.6%.

ICU non-survivors showed a higher baseline APACHE II score and SOFA score, higher age, heart rate (HR), Cumulative Vasopressor Index [21], arterial lactate level, serum creatinine and lower platelets count (Table 1).

Subsequently, patients were divided into two groups based on normal (≥ 2.6) or abnormal (< 2.6) baseline MFI. In comparison with patients with a normal MFI at baseline, patients with an abnormal MFI showed a higher ICU mortality (45 vs. 15.6%, *p* = 0.012) (Table 1), in-hospital mortality (55 vs. 28.6%, *p* = 0.035) and 90-day mortality (55 vs. 26%, *p* = 0.017). (Additional files 2, 3)

**Fig. 1:**
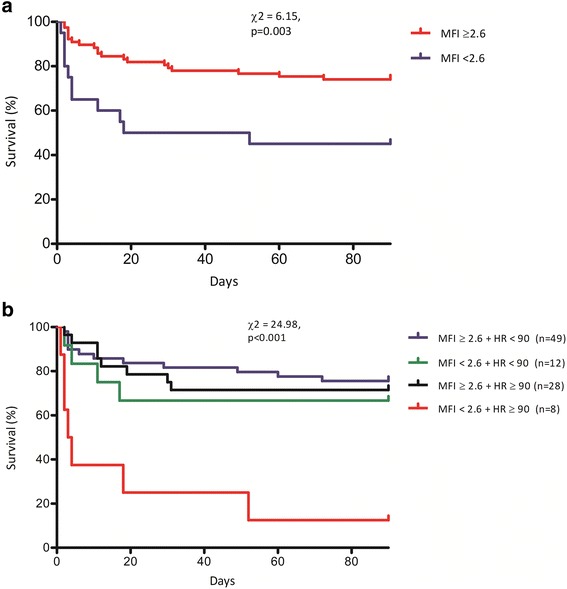
Kaplan–Meier survival analysis. **a** Represents two subgroups, separated by microvascular blood flow (MFI) < 2.6 versus MFI ≥ 2.6. **b** Represents four subgroups, separated by MFI with identical cut-off value and heart rate (HR) ≥ 90 versus < 90 bpm

Survival analysis, by Kaplan–Meier method, confirmed a significant difference between the two groups for 90-day mortality (Tarone–Ware *χ*^2^ = 6.15, *p* = 0.003) (Fig. 1a). In the binary logistic regression analysis, the presence of an abnormal MFI at baseline was associated with ICU mortality (OR 4.594 [95% CI 1.340–15.754], *p* = 0.015) independently of the APACHE II score (Table 2).

**Table 2:** Binary logistic regression analysis for ICU mortality

Variables	Odds ratio (95% CI)	*p* value
*ICU MORTALITY (abnormal MFI)*		
APACHE II score	1.204 (1.089–1.331)	< 0.001
MFI < 2.6	4.594 (1.340–15.754)	0.015
*ICU MORTALITY (abnormal MFI + tachycardia)*		
APACHE II score	1.191 (1.077–1.316)	0.001
MFI < 2.6 + tachycardia	10.732 (1.685–68.354)	0.012

In the upper model baseline, MFI abnormality was the independent variable. Model AUC 0.836 [0.747–0.904], Nagelkerke *R*^2^ 0.359, Hosmer and Lemeshow *χ*^2^ 4.733, *p* = 0.822. In the lower model, the presence of abnormal MFI plus tachycardia was the independent variable. Model AUC 0.836 [0.747–0.903], Nagelkerke *R*^2^ 0.374, Hosmer and Lemeshow *χ*^2^ 2.670, *p* = 0.914

*APACHE* acute physiologic and chronic health evaluation II, calculated in the first 24 h from ICU admission; *ICU* Intensive Care Unit; *MFI* Microvascular Flow Index. Abnormal MFI is defined as MFI < 2.6 for small vessels (diameter < 20 μm). Tachycardia is defined as a heart rate ≥ 90 bpm

The role of tachycardia in combination with an abnormal microcirculation was additionally tested. Patients were divided into four groups based on the presence of tachycardia (defined as the presence of an HR ≥ 90 beats per minute, bpm) [22–25] and/or MFI abnormality at the baseline. ICU mortality was significantly different between the four groups (overall *χ*^2^ = 12.76, *p* = 0.002). Survival analysis confirmed a significant difference between the groups in terms of 90-day mortality (Tarone–Ware *χ*^2^ = 24.98, *p* < 0.0001) with a survival rate as low as 12.5% among patients with tachycardia plus abnormal MFI (Fig. 1b). The combination of tachycardia and an abnormal MFI on day 1 was associated with an increased risk for ICU mortality (OR 10.732 [95% CI 1.685–68.354], *p* = 0.012) independently of the APACHE II score (Table 2).

### Integration of an abnormal microcirculation in a set of common hemodynamic variables

**Fig. 2:**
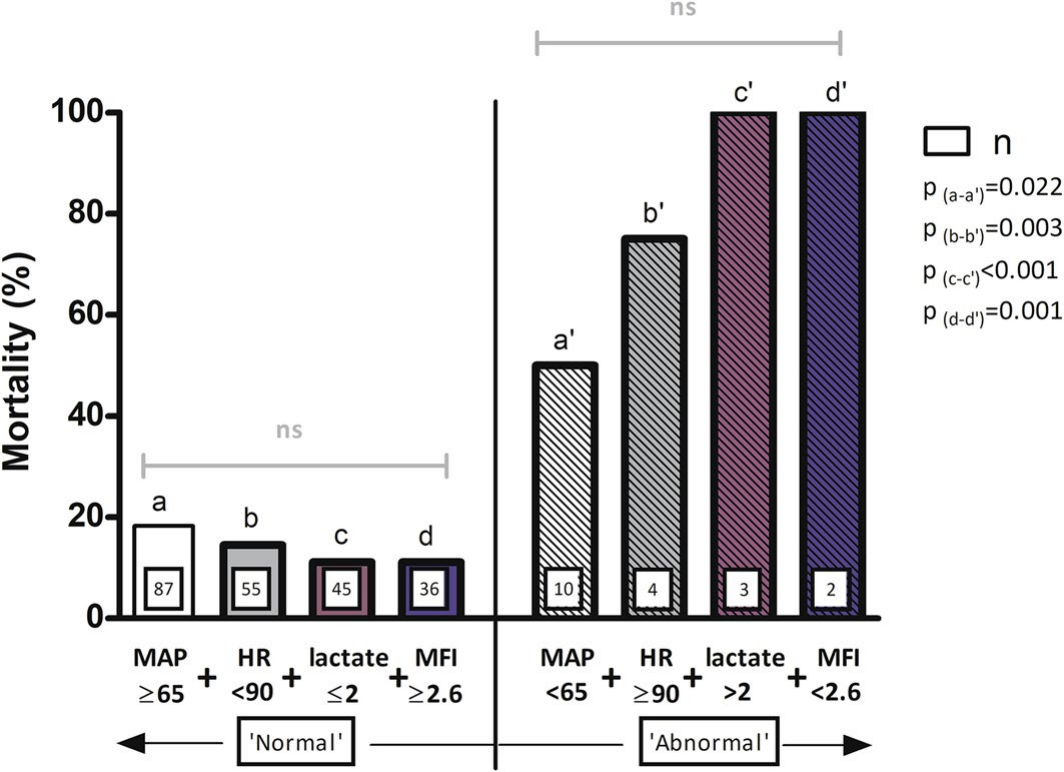
Prognostic model with stepwise inclusion of consecutive hemodynamic variables: mean arterial pressure (MAP) in mmHg, heart rate (HR) in bpm, (arterial) lactate in mmol/L and Microvascular Flow Index (MFI) in AU

In order to clarify the additional prognostic value of an abnormal microcirculation (MFI < 2.6) at baseline in a set of commonly available hemodynamic variables, i.e. mean arterial blood pressure (MAP), HR and lactate, we divided these variables into normal and abnormal: MAP ≥ 65 mmHg = normal, < 65 mmHg = abnormal; HR < 90 bpm = normal, ≥ 90 bpm = abnormal; and arterial lactate ≤ 2 mmol/L = normal, > 2 mmol/L = abnormal. In the first model, all variables were normal. A stepwise addition of each variable was associated with a non-significant reduction in ICU mortality (Fig. 2). In the second model, all variables were abnormal. A stepwise addition of each variable was associated with a non-significant increment in ICU mortality (Fig. 2). The comparison between the two models revealed in each step a significantly higher ICU mortality in the ‘abnormal’ model (Fig. 2).

### Microcirculatory longitudinal monitoring and outcome

The median duration of follow-up for each patient category was: 6 [3–12] days for trauma, 8 [3–14] days for neurologic, 5 [1–6] days for respiratory, 8 [3–11] days for septic and 4 [2–8] days for other patients.

**Fig. 3:**
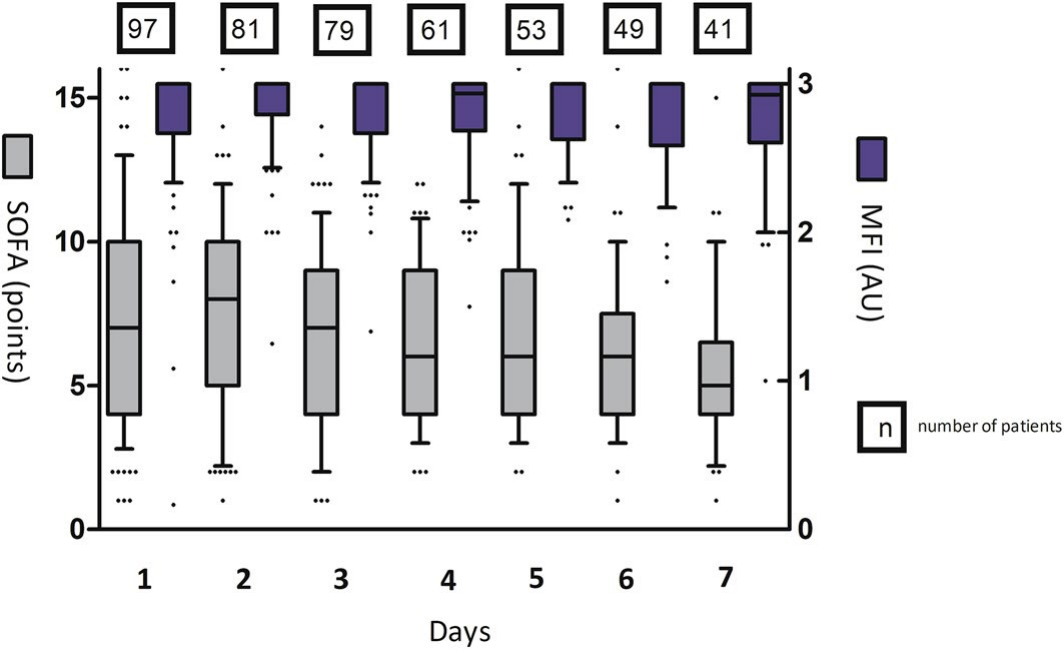
Evolvement over time of sequential organ failure assessment (SOFA) score and Microvascular Flow Index (MFI) in the first 7 days of ICU admission. Box and 10–90th percentile whisker plots with individual outliers

The total *incidence* of an abnormal microcirculation during the entire ICU stay was 55.7% (20.6% on day 1, 35.1% after day 1). Microcirculatory imaging was restricted to day 1 in ten patients (six died, four were discharged); missing data (SOFA and/or MFI) prevented further analysis in 19 patients. MFI and SOFA score over time are depicted in Fig. 3. Twenty-two patients showed an increment in MFI between days 1 and 2 (∆MFI (+)), 21 patients showed a reduction in MFI between days 1 and 2 (∆MFI (−)) and 25 remained indifferent. ∆MFI (+) was not associated with a significant reduction in SOFA score between days 2 and 3 (corresponding with the same time frame of MFI days 1 and 2) or mortality, as compared to patients with a ∆MFI (−). Any increase/decrease in MFI was considered relevant for this analysis.

Post hoc, patients were divided into four groups according to the timing of the presence of an abnormal MFI. Group 1: patients with a normal MFI on day 1 and later on (*n* = 36). Group 2: patients with a normal MFI on day 1 but with one or more episodes of an abnormal MFI later on (*n* = 34). Group 3: patients with an abnormal MFI on day 1 and a normal MFI later on (*n* = 6). Group 4: patients with an abnormal MFI on day 1 and one or more episodes of an abnormal MFI later on (*n* = 10). Mortality was significantly different across groups (*p* < 0.001). If an abnormal MFI was present on day one (groups 3 and 4), mortality was 6/16 (38%), whereas in patients with an abnormal MFI only after day 1 (group 2), ICU mortality was 2/34 (6%, *p* = 0.001), indicating a significant difference in prognostic value of an abnormal MFI on day 1 in comparison with an abnormal MFI after day 1.

## Discussion

This MicroDAIMON study is currently the largest prospective longitudinal observational study to describe the incidence of microcirculatory derangements among a mixed group of critically ill patients, offering a day-by-day follow-up. The incidence of baseline microcirculatory flow abnormalities was 20.6%, and more than half (55.7%) of the patients displayed an abnormal MFI in at least one observation during ICU stay. The main finding of this study is that in this mixed ICU population, an abnormal baseline MFI is independently associated with unfavourable outcome in terms of ICU, in-hospital and 90-day mortality. In addition, the contemporary presence of tachycardia showed an additive predictive power towards mortality in the survival analysis. However, the change of MFI over time was not associated with outcome, in terms of both organ failure (SOFA) and mortality. In contrast to an abnormal MFI on day 1, we could not associate an abnormal MFI after day 1 with unfavourable outcome. No associations were found between the other microvascular variables and outcome.

In 2015, the MicroSOAP study provided the first and largest database on the prevalence and the significance of the microcirculatory alterations in a heterogeneous ICU population, with a time-point observation across 36 ICUs worldwide [15]. The authors reported a prevalence of MFI abnormalities of 17%, using the same predefined cut-off value [15, 26]. This difference in reported percentage of MFI abnormalities can be explained by the difference in study design (longitudinal vs. point prevalence). Our data confirm previous observations, showing an important prognostic role of the microcirculation in various subsets of critically ill patients [4–9, 14]. In contrast to the existing literature, these findings extend the predictive value of early microcirculatory alterations towards 90-day mortality. Patients with an abnormal MFI at baseline showed an absolute risk of non-survival almost three times higher in comparison with patients with a normal MFI.

However, in the present study, routine day-by-day microcirculatory monitoring does not confirm previous observations. In 2004, Sakr et al. [11] introduced for the first time the concept of serial observations of microcirculation in a cohort of 49 patients with septic shock. In this highly selected group of patients, the persistence of microcirculatory alterations was associated with persistence of shock, development of multiple organ failure and mortality.

Conversely, ICU survivors showed early improvement of microcirculation. These data were confirmed by others [21]. Duranteau and colleagues observed in another selected cohort of 18 patients with traumatic haemorrhagic shock, early derangements of microcirculatory flow and vessel density, as well as its persistence, were able to predict a worse SOFA score after 96 h from ICU admission [5]. A possible explanation for this discrepancy may lie in the heterogeneous composition of our study population and in considerable differences in microcirculatory baseline abnormalities. Alternatively, microvascular alterations represent differences in underlying pathology between study populations. Careful selection of patients at risk may contribute to the prognostic power of microcirculatory observation. As of now, our data indicate that routine daily monitoring of the microcirculation in an unselected group of ICU patients is of limited prognostic value.

This study has several limitations. Although this study contains the largest reported database on day-by-day monitoring of the microcirculation in critically ill, it appears to have insufficient sample size to correlate differences in the evolution of microcirculatory conditions over time with clinically relevant endpoints (SOFA, mortality) also due to the considerable number of patients lost to follow-up, due to death/discharge. And although the independent predictive value of an abnormal MFI on day 1 was established, the integration of such variable in a model with more commonly used hemodynamic variables was clearly limited by the sample size as well. Further research is needed to establish the additive value of microcirculatory imaging on top of the existing hemodynamic variables. In addition, it is conceivable that other microvascular variables and different cut-off yield different results. We did not found any significant association between the other microcirculatory variables (TVD, PVD, PPV) and the outcome either on day 1 or in the following days. This could be explained by the fact that the MFI, especially if used as a dichotomous variable based on an a priori cut-off of 2,6, could have been the most sensitive variable to detect an association with the outcome in a such heterogeneous population which is expected to cause a “dilution effect” on the microcirculatory alterations. It is also possible that a vessel-by-vessel MFI calculation could have been more precise and provide different results depending on a more accurate evaluation of the capillary blood flow, especially in the presence of marked heterogeneity. In this respect, the burden of time-consuming offline analysis remains a major practical limitation for the study population sample size until the time of the development and full validation of automated analysis software. Real-time “eyeballing” the microcirculation by bedside assessment of MFI is a major advantage in the development of a bedside tool and showed good agreement with the gold standard offline analysis [27]. Post hoc analysis of our data confirmed 2.6 as the optimal cut-off for the discrimination between survivors and non-survivors. Finally, this was a pure observational study: patients were treated following the international guidelines and principles of good clinical practice, and clinicians had no information about the microcirculation during the study. Therefore, our study design is insufficient to draw conclusions on the applicability of microcirculatory monitoring as a tool to guide resuscitation. Even in the setting where there is an absence of additional prognostic information, derived from microcirculatory monitoring, the observation itself may contain valuable information about the underlying pathophysiologic mechanisms. For example, an increased lactate may adequately predict outcome, but does not reveal its underlying mechanism. Under these conditions, additional assessment of microvascular blood flow may not be useful to predict outcome, but may be helpful for the clinician to select the appropriate resuscitation strategy. Further research is needed to address this topic. Careful selection of subgroups and adequate timing remain of the essence in this process.

## Conclusions

This MicroDAIMON study provides data about incidence of microcirculatory alterations in a heterogeneous group of critically ill patients. Microcirculatory flow abnormalities at the baseline were independently associated with an increased risk of unfavourable outcome. Simultaneous presence of tachycardia enhanced this predictive value. However, neither the evolution of MFI over time nor the development or new abnormalities after day 1 was associated with organ function or mortality in our population with a sample size limitation. Further studies are needed to incorporate microcirculatory monitoring into a set of currently available hemodynamic variables and to establish its value as a tool to guide specific resuscitation strategies.

## Additional files

**Additional file 1.** Flow chart for patients’ recruitment. A schema to clarify the procedures for the patients’ recruitment for the study.

**Additional file 2.** Comparison between in-hospital survivors and non-survivors. The table illustrates the results of the univariable analysis for baseline clinical and microcirculatory variables between in-hospital survivors and non-survivors.

**Additional file 3.** Comparison between 90-day survivors and non-survivors. The table illustrates the results of the univariable analysis for baseline clinical and microcirculatory variables between 90-day survivors and non-survivors.

## Abbreviations

SDF: Sidestream Dark Field
ICU: Intensive Care Unit
SOFA: sequential organ failure assessment
MFI: Microvascular Flow Index
PPV: proportion of perfused vessels
TVD: total vessel density
PVD: perfused vessel density
SD: standard deviation
IQR: interquartile range
OR: odds ratio
CI: confidence interval
APACHE II: acute physiology and chronic evaluation score
HR: heart rate
MAP: mean arterial pressure
