# Supplementary figures and images for: MicroDAIMON study: Microcirculatory DAIly MONitoring in critically ill patients: a prospective observational study
Source: Ann Intensive Care. 2018 May 15;8:64. doi: 10.1186/s13613-018-0411-9 (PMC5953911; doi:10.1186/s13613-018-0411-9)

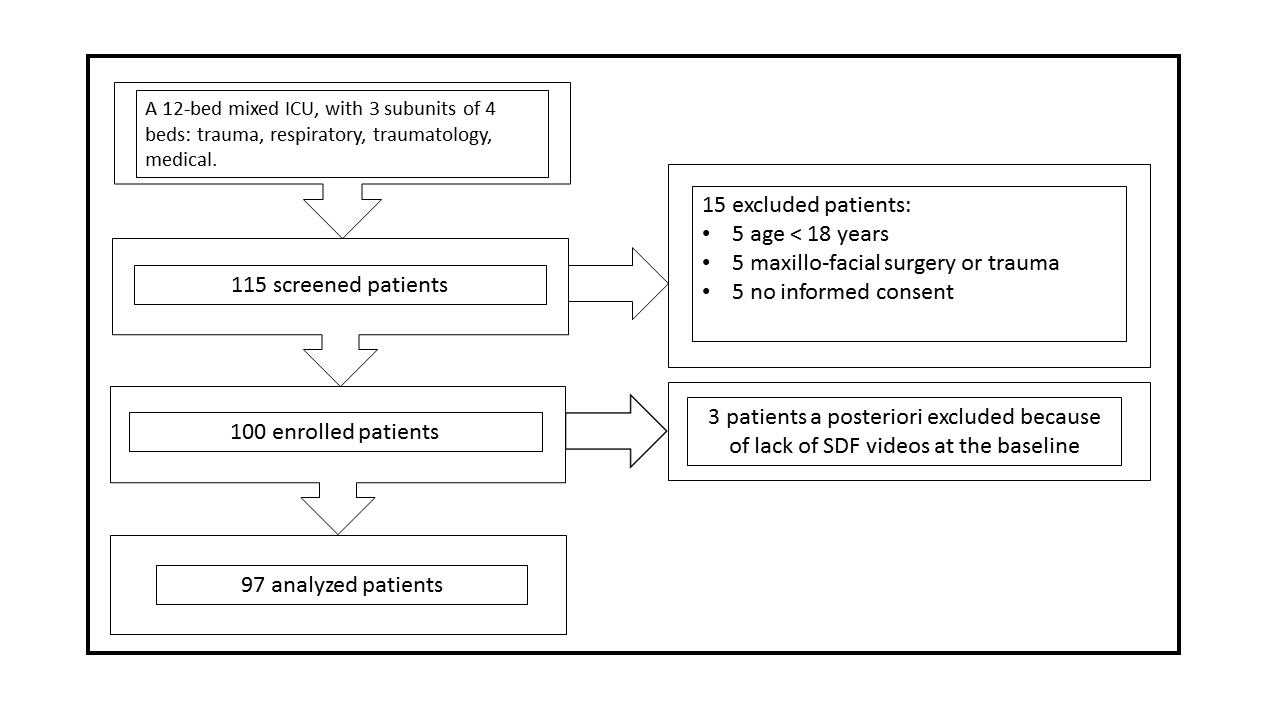

Supplement: Supplementary file 1 — Additional file 1. Flow chart for patients’ recruitment. A schema to clarify the procedures for the patients’ recruitment for the study. [file 13613_2018_411_MOESM1_ESM.tif]
