# Supplementary material for: MicroDAIMON study: Microcirculatory DAIly MONitoring in critically ill patients: a prospective observational study
Source: Ann Intensive Care. 2018 May 15;8:64. doi: 10.1186/s13613-018-0411-9 (PMC5953911; doi:10.1186/s13613-018-0411-9)
Supplement: Supplementary file 2 — Additional file 2. Comparison between in-hospital survivors and non-survivors. The table illustrates the results of the univariable analysis for baseline clinical and microcirculatory variables between in-hospital survivors and non-survivors. [file 13613_2018_411_MOESM2_ESM.docx]

**Supplemental Digital Content 2:** comparison between In-Hospital survivors and non-survivors.

| PATIENTS CHARACTERISTICS | *n* | *In-hospital survivors (64)* | *In-hospital non survivors (33)* | *p* |
| --- | --- | --- | --- | --- |
| Male gender (n, %) | 97 | 43 (67.2) | 21 (63.6) | 0.821 |
| Age (years, n) | 97 | 58[43-71] | 73[63-82] | **<0.001** |
| APACHE II (pts) | 97 | 14±7 | 21±6 | **<0.001** |
| SOFA (pts) | 97 | 5[3-9] | 10[8-13] | **<0.001** |
| ICU admission diagnosis, n (%) | 97 |  |  | 0.173 |
| Trauma | 37 | 29 | 8(21.6) |  |
| Neurologic | 21 | 14 | 7(33.3) |  |
| Respiratory | 11 | 7 | 4(36.4) |  |
| Sepsis | 9 | 5 | 4 (44.4) |  |
| Other | 19 | 9 | 10(52.6) |  |
| Heart rate (bpm) | 97 | 74[61-98] | 88[65-107] | 0.11 |
| Mean arterial pressure (mmHg) | 97 | 86±17 | 80±23 | 0.161 |
| Vasoactive drugs (treated) | 54 | 31(48.4) | 23(69.7) | 0.054 |
| Cumulative Vasopressor Index | 54 | 1[0-4] | 4[0-4] | **0.044** |
| Glasgow Coma Scale (pts) | 97 | 13[5-15] | 6[3-11] | **0.007** |
| Mechanical ventilation (n, %) | 97 | 71(93.7) | 33(100) | 0.163 |
| Peep (cmH_2_O) | 91* | 7[5-9] | 8[6-10] | 0.088 |
| Haemoglobin (g/dL) | 97 | 11.1±1.7 | 11±2 | 0.763 |
| White Blood Cells (nx10^3^/mmc) | 97 | 12[9-15.7] | 12.2[8.1-14.6] | 0.899 |
| Platelets (nx10^3^/mmc) | 97 | 169[111-201] | 126[93-182] | **0.048** |
| Creatinine (mg/dL) | 97 | 1[0.8-1.4] | 1.2[0.9-1.5] | 0.052 |
| Bilirubine (mg/dL) | 97 | 0.7[0.5-1] | 1[0.5-1.8] | **0.037** |
| PaO_2_ (mmHg) | 97 | 149[114-178] | 134[91-158] | 0.103 |
| Arterial lactates (mmol/L) | 97 | 1.2[0.9-1.67] | 1.6[1.4-3.5] | **<0.001** |
| ScvO_2_ (%) | 59** | 78.8[71.7-83.8] | 75.4[66.1-80.5] | 0.164 |
| Comorbidities, n(%)   - Obesity - Hypertension - Diabetes mellitus - Hypercholesterolemia - Malignancy - Vascular disease - Cardiomiopathy - COPD - Renal insufficiency |  |  |  | ns |
| MICROCIRCULATORY VARIABLES: |  |  |  |  |
| TVD (small) (mm/mm^2^) | 97 | 20.1[16.9-22.6] | 20.9[17.4-22.8] | 0.817 |
| PVD (small) (mm/mm^2^) | 97 | 19.1±4 | 19.8±5 | 0.52 |
| De Backer score (n/mm) | 97 | 11.7±2 | 12.3±2 | 0.152 |
| PPV (small) (%) | 97 | 98[94.2-99.6] | 98.6[97-100] | 0.199 |
| MFI (small) (AU) | 97 | 3[2.8-3] | 3[2.5-3] | 0.624 |
| HI (small) | 97 | 0[0-0.2] | 0[0-0.3] | 0.982 |
| Abnormal MFI (n,%) | 97 | 9(16.4) | 11(50) | **0.035** |

Data are presented as mean ± SD or as median [IQR] unless stated otherwise. APACHE Acute Physiologic And Chronic Health Evaluation II, calculated over the first 24 hours from ICU admission;

SOFA Sequential Organ Failure Assessment, calculated over the first 24 hours from ICU admission. CVI Cumulative Vasopressor Index; ICU Intensive Care Unit; COPD, Chronic Obstructive Pulmonary Disease. TVD Total Vessel Density; PVD Perfused Vessel Density; PPV Proportion of Perfused Vessel; HI Heterogeneity Index; MFI Microvascular Flow Index. Abnormal MFI is defined as MFI < 2,6. Cut off value for small vessels diameter < 20 μm.
