# Supplementary material for: MicroDAIMON study: Microcirculatory DAIly MONitoring in critically ill patients: a prospective observational study
Source: Ann Intensive Care. 2018 May 15;8:64. doi: 10.1186/s13613-018-0411-9 (PMC5953911; doi:10.1186/s13613-018-0411-9)
Supplement: Supplementary file 3 — Additional file 3. Comparison between 90-day survivors and non-survivors. The table illustrates the results of the univariable analysis for baseline clinical and microcirculatory variables between 90-day survivors and non-survivors. [file 13613_2018_411_MOESM3_ESM.docx]

**Supplemental Digital Content 3:** comparison between 90-day survivors and non-survivors.

| PATIENTS CHARACTERISTICS | *n* | *90-day survivors (66)* | *90-day non survivors (31)* | *p* |
| --- | --- | --- | --- | --- |
| Male gender (n, %) | 97 | 45 (68.2) | 19 (63.6) | 0.502 |
| Age (years, n) | 97 | 58[43-71] | 73[63-81] | **<0.001** |
| APACHE II (pts) | 97 | 14±7 | 21±6 | **<0.001** |
| SOFA (pts) | 97 | 5[4-9] | 10[8-13] | **<0.001** |
| ICU admission diagnosis, n (%) | 97 |  |  | 0.112 |
| Trauma | 37 | 30 | 7(18.9) |  |
| Neurologic | 21 | 15 | 6(28.6) |  |
| Respiratory | 11 | 7 | 4(36.4) |  |
| Sepsis | 9 | 5 | 4 (44.4) |  |
| Other | 19 | 9 | 10(52.6) |  |
| Heart rate (bpm) | 97 | 74[61-95] | 88[69-107] | 0.086 |
| Mean arterial pressure (mmHg) | 97 | 86±17 | 81±23 | 0.264 |
| Vasoactive drugs (treated) | 54 | 31(46.9) | 23(74.2) | **0.016** |
| Cumulative Vasopressor Index | 54 | 0[0-4] | 4[0-4] | **0.011** |
| Glasgow Coma Scale (pts) | 97 | 13[5-15] | 6[3-11] | **0.011** |
| Mechanical ventilation (n, %) | 97 | 61(92.4) | 31(100) | 0.174 |
| Peep (cmH_2_O) | 91* | 7[6-8] | 8[7-10] | **0.037** |
| Haemoglobin (g/dL) | 97 | 11.1±1.7 | 10.9±2.1 | 0.724 |
| White Blood Cells (nx10^3^/mmc) | 97 | 11.5[8.9-15.2] | 12.5[8.4-14.6] | 0.76 |
| Platelets (nx10^3^/mmc) | 97 | 168[116-200] | 122[92-187] | **0.049** |
| Creatinine (mg/dL) | 97 | 1[0.8-1.4] | 1.2[1-1.5] | **0.023** |
| Bilirubine (mg/dL) | 97 | 0.7[0.5-1] | 1[0.5-1.7] | **0.041** |
| PaO_2_ (mmHg) | 97 | 149[115-177] | 122[90-158] | 0.081 |
| Arterial lactates (mmol/L) | 97 | 1.2[0.9-1.73] | 1.6[1.4-3.6] | **<0.001** |
| ScvO_2_ (%) | 59** | 79[71.9-83.5] | 75.2[65.2-80.8] | 0.127 |
| Comorbidities, n(%)   - Obesity - Hypertension - Diabetes mellitus - Hypercholesterolemia - Malignancy - Vascular disease - Cardiomiopathy - COPD - Renal insufficiency |  |  |  | ns |
| MICROCIRCULATORY VARIABLES: |  |  |  |  |
| TVD (small) (mm/mm^2^) | 97 | 20.1[17.1-22.6] | 20.9[17.2-22.7] | 0.817 |
| PVD (small) (mm/mm^2^) | 97 | 19.1±4 | 19.7±5 | 0.595 |
| De Backer score (n/mm) | 97 | 11.7±2 | 12.3±2 | 0.184 |
| PPV (small) (%) | 97 | 98[94.5-99.7] | 98.6[96.7-100] | 0.28 |
| MFI (small) (AU) | 97 | 3[2.8-3] | 3[2.4-3] | 0.378 |
| HI (small) | 97 | 0[0-0.2] | 0[0-0.3] | 0.731 |
| Abnormal MFI (n,%) | 97 | 9(13.6) | 11(35.5) | **0.017** |

Data are presented as mean ± SD or as median [IQR] unless stated otherwise. APACHE Acute Physiologic And Chronic Health Evaluation II, calculated over the first 24 hours from ICU admission;

SOFA Sequential Organ Failure Assessment, calculated over the first 24 hours from ICU admission. CVI Cumulative Vasopressor Index; ICU Intensive Care Unit; COPD, Chronic Obstructive Pulmonary Disease. TVD Total Vessel Density; PVD Perfused Vessel Density; PPV Proportion of Perfused Vessel; HI Heterogeneity Index;. MFI Microvascular Flow Index. Abnormal MFI is defined as MFI < 2,6. Cut off value for small vessels diameter < 20 μm.
